# Incidence of unplanned intensive care unit admission following surgery and associated factors in Amhara regional state hospitals

**DOI:** 10.1038/s41598-022-24571-1

**Published:** 2022-11-22

**Authors:** Tikuneh Yetneberk, Meseret Firde, Abebe Tiruneh, Yewlsew Fentie, Mequanent Tariku, Gashaw Mihret, Jolene Moore

**Affiliations:** 1 Department of Anesthesia, Debre Tabor University, Debre Tabor, Ethiopia; 2 Department of Gynecology and Obstetrics, Debre Tabor University, Debre Tabor, Ethiopia; 3 School of Medicine, Debre Tabor University, Debre Tabor, Ethiopia; 4 School of Medicine, Medical Sciences and Nutrition, University of Aberdeen, Aberdeen, UK

**Keywords:** Health care, Health services

## Abstract

Unplanned postoperative critical care admission poses a potential risk to patients and places unanticipated pressure on clinical services and it has become an important parameter to assess patient safety in perioperative services. This study was aimed to determine the incidence of unplanned intensive care unit admission following surgery and the associated factors. A multi-center cross-sectional study was conducted on postoperative patients admitted to the ICU of three hospitals located in the Amhara region. Data were collected via a structured survey tool and analyzed using SPSS version 23 software with binary logistic regression analysis. The statistical significance to identify patient, anesthetic and surgical related factors in the preoperative, intraoperative, and postoperative period was < 0.05 for multivariable regression with a 95% confidence interval. Predominantly patients were admitted to the ICU in an unplanned manner. ASA status, preoperative hemoglobin (Hgb) level, intraoperative estimated blood loss, and adverse events occurring in the operating room were significantly associated with intensive care unit admission following surgery. Patients who had a low preoperative Hgb value were 35.1 times more likely to be admitted to the intensive care unit in an unplanned manner compared with their counterparts [(Adjust odds ratio (AOR) 35.16; CI 12.82, 96.44)]. Patients with ASA II and III were 19.4 and 16.2 times more likely to be admitted to ICU in an unplanned way compared to patients who had ASA I physical status [(AOR 51.79; CI 8.28, 323.94) (AOR 67.8 CI 14.68, 313.53)]. Unplanned ICU admission after surgery was high in this study, suggesting poor perioperative planning, risk stratification, and optimization of patients.

## Introduction

An intensive care unit (ICU) is a place where comprehensive life-saving care is provided for critically ill patients^1^. Managing critically ill patients in an ICU requires significant human, infrastructural, and financial resources^2^. The decision to admit a patient to the ICU, high dependency unit, or the post-anesthetic care unit (PACU) is usually dependent on the surgical procedure, the patient’s comorbidities, institutional policy as well as the availability of beds^3^. Numerous ICU admissions occur after surgery and anesthesia; however, not all postoperative ICU admissions are planned or anticipated before the scheduled case^4^. Post-operative ICU admission is frequently required in an unplanned manner during the perioperative period, due to complications related to surgery, anesthesia, or underlying illness^5^.

Compared with planned ICU admission, unplanned ICU admission is associated with a significantly higher risk of death beyond the expected consequences of comorbidities, age, type of surgery, and emergency status^2^. It has long been recognized that admissions to the ICU can be due to complications caused by patient care rather than by patient illness^6^; This is particularly true when ICU admissions occur in an unexpected or unplanned manner^4^. Thus, unplanned admission to the ICU from the operating room, PACU, recovery room, or surgical ward has been considered to be an important clinical indicator and measure of the quality of anesthetic and surgical care in the surgical patient population^7,8^.

Despite planned ICU admission being a standard of care following certain surgical procedures or in high-risk patients for decades, it is not always possible, particularly in low or middle-income countries^9^. Where ICU services and other resources are limited, unplanned ICU admission may further constrain the resources^9^. Furthermore, unplanned ICU admission is associated with increased mortality and increased length of hospital stay^8^.

### Objective

To determine the incidence of unplanned ICU admission following surgery in Amhara regional state hospitals over 6 months in 2020/2021, and identify associated factors.

## Methods

A prospective multicenter cross-sectional study was conducted in three hospitals in the Amhara region of Northern Ethiopia over—6 months from October 2020 to March 2021. Included hospitals were: Debre Tabor's comprehensive specialized hospital, Felege Hiwot comprehensive specialized hospital, and Tibebe Ghion specialized and comprehensive hospital. These hospitals, located in the cities of Bahir Dar and Debre Tabor, serve as specialized referral facilities to the surrounding region and population and offer a broad range of surgical services. Those hospitals aided for more than 15 million people of the Amhara region. Approximately more than 20,000 surgeries performed annually in the hospitals.

The intensive care units are organized in a thorough manner, providing critical care services for both surgical and medical cases in a single unit. Anesthetists, internists, and nurses with formal ICU training as well as those with short-term (less than a month) ICU training are included in the ICU staffing. Anesthetists and internists receive formal intensive care training as part of their specialty training (i.e. during masters or residency training). The intensive care unit typically had a small capacity and staff.

Surgical patients who were admitted to ICU following surgery and anesthesia at these facilities were assessed for elgibility to the study. Unplanned ICU admission is defined as a surgical case in which ICU admission was determined intra- or postoperatively within 5 days following surgery and anesthesia. Planned ICU admission is defined as a surgical case in which the ICU reservation was made before surgery and anesthesia^1,4^. Patients who were already admitted to the ICU before surgery and surgical patients who were admitted to the ICU 5 or more days after the surgical procedure were excluded. Patients who were referred to the ICU from other hospitals after any surgical procedures were also excluded from the study.

All consecutive patients who fulfilled the inclusion criteria and were admitted to the ICU following surgery were recruited until the intended sample size was achieved. The sample size was determined by using single population proportion formula based on the following assumption, since there is no previous study conducted in this topic in Ethiopia, we assumed that the prevalence of unplanned intensive care unit admission following surgery is 50% with 95% confidence interval, 5% margin of error, the sample size is calculated as follow.

$${\text{n }} = \, ({\text{Z}}_{{\alpha /{2}}} )^{{2}} \,{\text{P }}\left( {{1} - {\text{P}}} \right)/{\text{d}}^{{2}} ,$$

$${\text{n}} = \, \left( {{1}.{96}} \right)^{{2}} \times \, 0.{5}\left( {{1} - 0.{5}} \right)/ \, \left( {0.0{5}} \right)^{{2}} ,$$

$${\text{n}} = {385} .$$

The total sample size after including 10% non-response rate will be:

$${\text{n}} = {385} + {39,}$$

$${\text{n}} = {424}\,{\text{participants}}{.}$$

Socio-demographic variables, anesthesia-related factors, and surgery-related factors were studied. A paper-based survey tool was developed in the English language, for completion by ICU staff at the facilities, aided by a review of patient medical records. Following survey completion, data was entered into Epidata version 4.2. Software by the research team and exported to SPSS version 23 statistical software for further analysis. Descriptive statistics are presented as mean ± standard deviation for normally distributed data, and non-normally distributed data are presented as median and interquartile ranges. Results are presented in text and tabular format.

Binary logistic regression analysis was utilized to identify independent risk factors for postoperative unplanned ICU admission. The association between independent factors and outcome variables was determined by chi-squared test, bivariable and multivariable logistic regression. Crude and adjusted odds ratios with 95% confidence intervals were used for estimation of association strength. Statistical significance was determined as P < 0.2 for bivariable and P < 0.05 for multivariable regression.

The research was taken ethical approval letter from Debre Tabor University College of Health Sciences Ethical Review committee (Ref no.183/2013) and written informed consent was taken from each study participant or their next of kin. The research was adhered with the declaration of Helsinki.

### Ethics approval and consent to participate

In order to keep the ethical soundness of the research, an ethical approval letter was obtained from the Institutional Review Board (IRB) of Debre Tabor University. Verbal and written consent was also secured before data collection.

## Result

### Characteristics of study participants

A total of 421 postoperative patients were admitted to ICUs at the three study facilities during the study period. The mean age of patients was 39.79 ± 12.17 years, the majority (n = 241; 57.2%) were female, were ASA I physical status, and received general anesthesia (92.2%) (Table 1).

**Table 1:** Characteristic of patient admitted to intensive care unit following surgery, 2021.

Variables	Category	Frequency	Percent
Gender	Male	180	42.8
	Female	241	57.2
Age	20–30 years	148	32.8
	31–40 years	114	24.7
	Greater than 41 years	159	42.5
ASA status	ASA I	195	46.3
	ASA II	100	23.8
	ASA III	93	22.1
	ASA IV	33	7.8
Type of surgery	Elective	46	10.9
	Emergency	375	89.1
Primary anesthesia type	General anesthesia	388	92.2
	Neuraxial anesthesia	33	7.8
Surgical specialty	General	167	40.1
	ENT	47	11.2
	Orthopedics	11	2.6
	Neuro	80	18.8
	Gyn/OBS	47	11.2
	Hepatobiliary	12	2.6
	Gastrointestinal	57	13.5

Most adverse events (73.2%) resulting in ICU admission occurred in the operating room. The majority of patients (54.6%) admitted to the ICU postoperatively had low preoperative hemoglobin (less than 12 g/dL) values. Predominantly patients were admitted to the intensive care unit in an unplanned manner (Table 2).

**Table 2:** Perioperative factors in patients admitted to ICU following surgery, 2021.

Variable	Category	Frequency	Percent
The location where an adverse event occurred	Operating room	308	73.2
	Recovery room	45	10.7
	Surgical ward	68	16.2
Manner of ICU admission	Planned	79	18.8
	Unplanned	342	81.2
BMI (kg/m^2^)	Low	11	2.6
	Normal	330	78.4
	High	80	19
Preoperative Hgb level	Low (< 12 g/dL)	230	54.6
	Normal	191	45.4
Duration of surgery (h)	Less than 2 h	115	27.3
	Greater than or equal to 2 h	306	72.7
Duration of anesthesia (h)	Less than 2 h	58	13.8
	Greater than or equal to 2 h	363	86.2
Experience of the anesthetist	Less than 5 years	57	13.5
	Greater than or equal to 5 years	364	86.5
Experience of the surgeon	Less than 5 years	45	10.7
	Greater than or equal to 5 years	376	89.3
Estimated blood loss (mL)	Less than 750 mL	226	53.7
	Greater than or equal to 750 mL	195	46.3

*ICU* Intensive care unit, *BMI* Body mass index (normal = 18.5–24.9 kg/m^2^, low < 18.5 kg/m^2^, high > 24.9 kg/m^2^), *ml* milliliter, *min* minute, *g/dL* grams per deciliter, *Hgb* Hemoglobin (normal = 12–15 g/dL, low < 12 g/dL).

Multivariate logistic regression analysis revealed ASA status, preoperative Hgb level, intraoperative estimated blood loss, and adverse events occurring in the operating room were significantly associated with ICU admission following surgery and anesthesia.

Patients with a low preoperative hemoglobin value were 35.1 times more likely to be admitted to the ICU in an unplanned manner compared with their counterparts (AOR 35.16; CI 12.82, 96.44) (Table 3). Patients with a physical status of ASA II and III were 19.4 and 16.2 times more likely to be admitted to ICU in an unplanned way compared to patients who had ASA I physical status [(AOR 51.79; CI 8.28, 323.94) (AOR 67.8 CI 14.68, 313.53)] (Table 3).

**Table 3:** Factors associated with intensive care unit admission following surgery, 2021.

Variables	Category	ICU admission		COR (95% CI)	AOR (95% CI)
		Planned	Unplanned		
		(N = 79)	(N = 342)		
Gender	Male	33	147	1.05 (0.64, 1.72)	
	Female	46	195	1	
Age	20–30 years	11	127	0.93 (0.70, 1.24)	
	31–40 years	46	58	0.56 (0.65, 1.32)	
	≥ 41 years	22	157	1	
ASA status	ASA I	34	161	1	1
	ASA II	11	89	19.47 (4.2, 21.3)*	51.8 (8.28, 323.94)**
	ASA III	12	81	16.2 (6.21, 42.14) *	67.8 (14.68, 313.53)**
	ASA IV	22	21	13.5 (5.25, 34.7)*	12.23 (15.2, 25.23)
Type of surgery	Elective	0	46	1	1
	Emergency	79	296	2.3 (2.45, 5.62)*	4.5 (24.3, 40.56)
Primary anesthesia type	GA	68	320	2.35 (1.09, 5.08)*	3.04 (0.67, 13.69)
	NA	11	22	1	1
Presence of comorbidity	Yes	45	102	3.1 (1.88, 5.14)*	2.34 (0.45, 3.42)
	No	34	240	1	1
The location where the adverse event occurred	Operation room	45	263	5.88 (3.3, 10.34)*	10.4 (3.84, 26.26)**
	Recovery room	0	45	2.3 (2.3, 5.23)	1
	Surgical ward	34	34	1	
BMI (kg/m^2^)	Low	0	11	3.23 (2.3, 11.23)	
	Normal	79	251	1	
Estimated blood loss (mL)	Less than 750 mL	34	192	1	1
	Greater than or equal to 750 mL	45	150	1.69 (1.03, 2.77)*	12.8 (3.77, 38.64)**
Duration of surgery (min)	Less than 2 h	23	92	1	
	Greater than or equal to 2 h	56	250	1.11 (0.65, 1.29)	
Duration of anesthesi a (min)	Less than 2 h	12	46	1	
	Greater than or equal to 2 h	67	296	1.15 (0.57, 2.29)	
Experience of the anesthetist	Less than 5 years	11	46	0.96 (0.47, 1.95)	
	Greater than or equal to 5 years	68	296	1	
Experience of the surgeon	Less than 5 years	10	35	1.27 (0.6, 2.69)	
	Greater than or equal to 5 years	69	307	1	
Preoperative Hgb level	Low (< 12 g/dL)	68	168	6.86 (3.51, 13.44)*	35.16 (12.82, 96.44)**
	Normal	11	180	1	

*ASA* American Society of Anesthesiologists, *GA* general anesthesia, *NA* neuraxial anesthesia, *BMI* Body mass index, *COR* crude odds ratio, *AOR* adjusted odds ratio, *CI* confidence interval, *(p-value < 0.2) Crude odds ratio, **(P value < 0.05) adjusted odds ratio, *N* number, *Hgb* hemoglobin, *g/dL* grams per deciliter.

Adverse events that occurred in the operating room were 10.4 times more likely to be a reason for unplanned ICU admission after surgery compared to an adverse events that happened outside the operating room (AOR 10.4; CI 3.84, 26.26) (Table 3). Patients who had more than 750 mL of intraoperative estimated blood loss were 12.8 times more likely to be admitted to the intensive care unit after surgery in an unplanned way compared to their counterparts (AOR 12.8; CI 3.77, 38.64) (Table 3).

## Discussion

In this study, 81.2% of patients were admitted to the intensive care unit in an unplanned manner following surgery and anesthesia. Globally, 43.5% of surgical patients are admitted to the critical care unit post-surgically in an unplanned way, far lower than the prevalence in the present study^10^. The probable justification for this discrepancy may be the former study is a global pooled result study that incorporates a wide range of perioperative care facilities with varying set-ups. Furthermore, the health care facilities comprised in this study did not have a clear intensive care unit admission guideline/policy which may escalate the prevalence of unplanned ICU admission following surgery.

The present study revealed that the likelihood of unplanned postoperative ICU admission in three facilities in the Amhara region of Ethiopia was increased in patients who were classified as ASA physical status II or III, and in cases with a low preoperative hemoglobin value, greater than 750 mL of intraoperative estimated blood loss, and cases performed under general anesthesia. This is in keeping with the findings of other studies^2,11,12^.

In this study, we found that adverse events that occur in the operating room were more commonly a reason for unplanned ICU admission postoperatively than adverse events occurring in other post-operative locations. This further supports studies from Nigeria, Belgium, and Australia where anesthetic and surgical-related adverse events were the reason for most unplanned ICU admissions^7,13,14^.

Whilst intraoperative adverse events and higher volume of blood loss may be considered unpredictable, and it may be expected that higher ASA status, indicating poorer functional performance pre-operatively, would be associated with increased risk of ICU admission, it could be argued that careful attention to pre-operative assessment and patient optimization may have helped to prevent unplanned admission in a proportion of these cases. This is a pertinent consideration for those patients with a low preoperative haemoglobin, in whom this could potentially have been detected and managed prior to surgery. These patients had a significantly higher risk of unplanned ICU admission in this study. However, this group of patients were not further analysed to determine whether preoperative haemoglobin optimization strategies would have been feasible.

In addition, detailed analysis of patient comorbidies and type of adverse event which occurred was not analysed. We recognize these as limitations of the study and an area of potential future work. In this study, the outcome of patients after postoperative ICU admission was also not studied and would be an interesting component of further studies. We guarantee that every piece of information included in this study was carefully gathered.

## Conclusion

Unplanned ICU admission after surgery was high, indicating poor perioperative care, risk stratification, and optimization of patients. We recommended strengthening pre-operative assessment, patient optimization and perioperative care services in the facilities studied and the wider region.

## Data Availability

All data generated or analysed during this study are included in this article.

## Abbreviations

AOR: Adjusted odds ratio
ASA: American Society of Anesthesiologists
COR: Crude odd ratio
ICU: Intensive care unit
PACU: Post anesthesia care unit
UIA: Unplanned intensive care unit admission
